# Review of genetic diversity in Bactrian camel (*Camelus bactrianus*)

**DOI:** 10.1093/af/vfac027

**Published:** 2022-08-12

**Authors:** Liang Ming, Dalai Siren, Surong Hasi, Tuyatsetseg Jambl, Rimutu Ji

**Affiliations:** Key Laboratory of Dairy Biotechnology and Engineering, Ministry of Education, College of Food Science and Engineering, Inner Mongolia Agricultural University, 010018, Hohhot, China; Key Laboratory of Dairy Biotechnology and Engineering, Ministry of Education, College of Food Science and Engineering, Inner Mongolia Agricultural University, 010018, Hohhot, China; Key Laboratory of Clinical Diagnosis and Treatment Technology in Animal Disease, Ministry of Agriculture, College of Veterinary Medicine, Inner Mongolia Agricultural University, 010018, Hohhot, China; China-Mongolia Joint Laboratory for Biomacromolecule Research, Ulaanbaatar, Mongolia; Key Laboratory of Dairy Biotechnology and Engineering, Ministry of Education, College of Food Science and Engineering, Inner Mongolia Agricultural University, 010018, Hohhot, China; Inner Mongolia Institute of Camel Research, 737300, Alax, Inner Mongolia, China

**Keywords:** Bactrian camel, genetic diversity, genome

**Implications**

- Research on the origin and domestication of Bactrian camel is helpful to understand the process of animal evolution and plays an important role in protecting the genetic diversity of Bactrian camel populations.
- New advances in research of the origin and domestication of Bactrian camel have been acquired by the rapid development of next-generation sequencing technology, molecular genetics, and bioinformatics.
- An overview given about the distribution, breeds, evolutionary history, and domestication, as well as the genetic diversity based on mtDNA, nuclear, and whole-genome sequencing of Bactrian camel populations.

## Introduction

**Figure 1.**
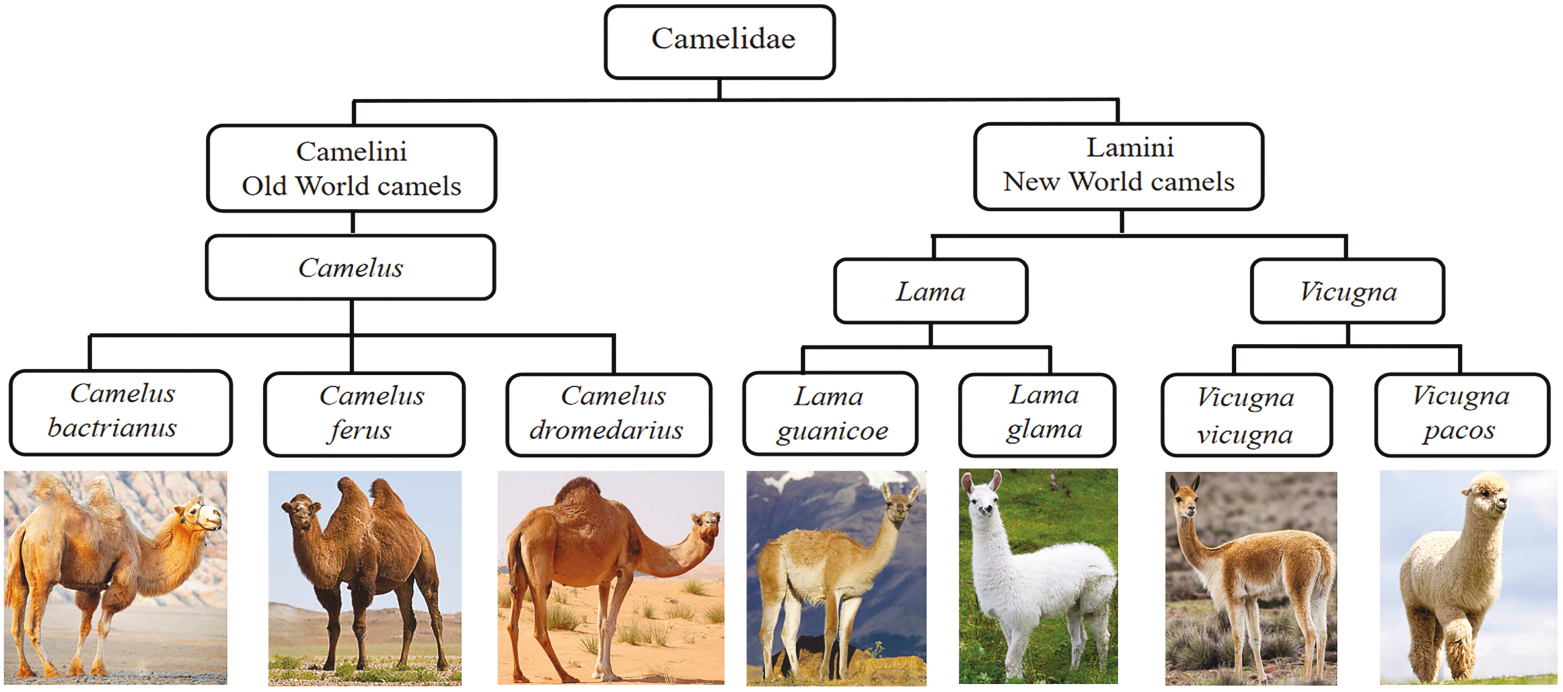
Taxonomy of the family Camelidae.

Bactrian camels (*Camelus bactrianus*) adapt to the specific weather conditions of both desert and semi-desert regions and provide a wide range of useful products to Gobi Desert rural communities, including meat, milk, and wool. In animal taxonomy, the Bactrian camel belongs to Animalia, Chordata, Mammalia, Placentalia, Artiodactyla, Tylopoda, and Camelidae. The family of Camelidae consists of the tribes Camelini (Old World camels) and Lamini (New World camels). The Camelini includes the *Camelus* genus, and the Lamini is divided into two genera, *Lama* and *Vicugna*. The genus *Camelus* includes three species, domesticated single-humped camel (dromedary, *Camelus dromedarius*), distributed in the arid deserts of North Africa, East Africa, and the Arabian Peninsula; domesticated two-humped camel (Bactrian camel, *Camelus bactrianus*), found in cooler areas of Asia (Faye and Konuspayeva, 2012), and the wild two-humped camel (*Camelus ferus*), considered critically endangered by the International Union for Conservation of Nature (Hare, 2008). The tribe Lamini comprises two domestic species of llama (*Lama glama*) and alpaca (*Vicugna pacos*) and two wild species of Guanaco (*Lama guanicoe*) and vicuña (*Vicugna vicugna*) (Saipolda, 2005; Wu et al., 2014). The classification of the family Camelidae is given in Figure 1.

To survive and live in harsh conditions, camels developed many special abilities and attributes. They can adapt to desert and semi-desert conditions—cold, hot, arid, poor grazing, and even food shortages, and can withstand extreme thirst and hunger for a long time due to sufficient energy in their humps and abdomen (Emmanuel and Nahapetian, 1980). The body temperature of the camel may vary from 34°C to 41°C throughout the day (Bouâouda et al., 2014). Different from other mammals, camels’ red blood cells are oval, which is conducive to water storage (Warda et al., 2014). They can consume diets toxic to other mammals due to their special metabolic pathways and unique detoxification capabilities (Hasi et al., 2018). Due to the extreme living environment in desert areas, they feed on Asteraceae and Chenopodiaceae, which have a high salt content, and herbaceous plants, bush, and cacti, which are rich in fiber but contain very little protein. Blood glucose levels in camels are twice those of other ruminants (Al-Ali et al., 1988). In addition, camelids are the only mammals that can produce heavy-chain antibodies (HCAbs), which lack the light chain, in contrast to conventional antibodies (Hamers-Casterman et al., 1993).

In this review, we aim to provide a comprehensive summary of the distribution, breeds, evolutionary history, and domestication, as well as the genetic diversity based on mitochondrial DNA (mtDNA), nuclear, and whole-genome sequencing of Bactrian camel populations.

## Bactrian Camel Distribution and Popular Breeds

**Figure 2.**
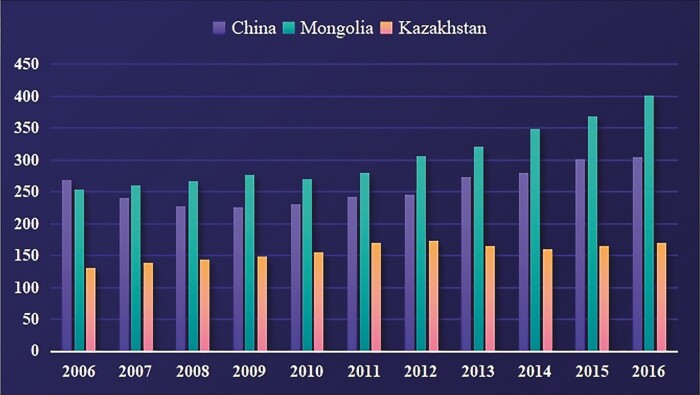
The growth trend of Bactrian camels in China, Mongolia, and Kazakhstan from 2006 to 2016. Reprinted from the Material of Jirimutu et al. (2022).

Bactrian camel numbers and distribution vary from region to region. The ecological geographical distribution is in the transition from grasslands to desert areas; the higher the degree of desertification, the more Bactrian camels there are. Although the service value of Bactrian camels has substantially decreased, the trend of nurturing camels in the desert is increasing at the global level. Of the entire Bactrian camel population, more than 90% are found in China, Mongolia, and Kazakhstan. The Bactrian camel population was estimated to be 381,000 in China, 401,000 in Mongolia, and 170,000 in Kazakhstan in 2016 according to the Food and Agriculture Organization (FAO) statistics. The growth trend of Bactrian camels in China, Mongolia, and Kazakhstan from 2006 to 2016 is shown in Figure 2 (Jirimutu et al., 2022).

**Figure 3.**
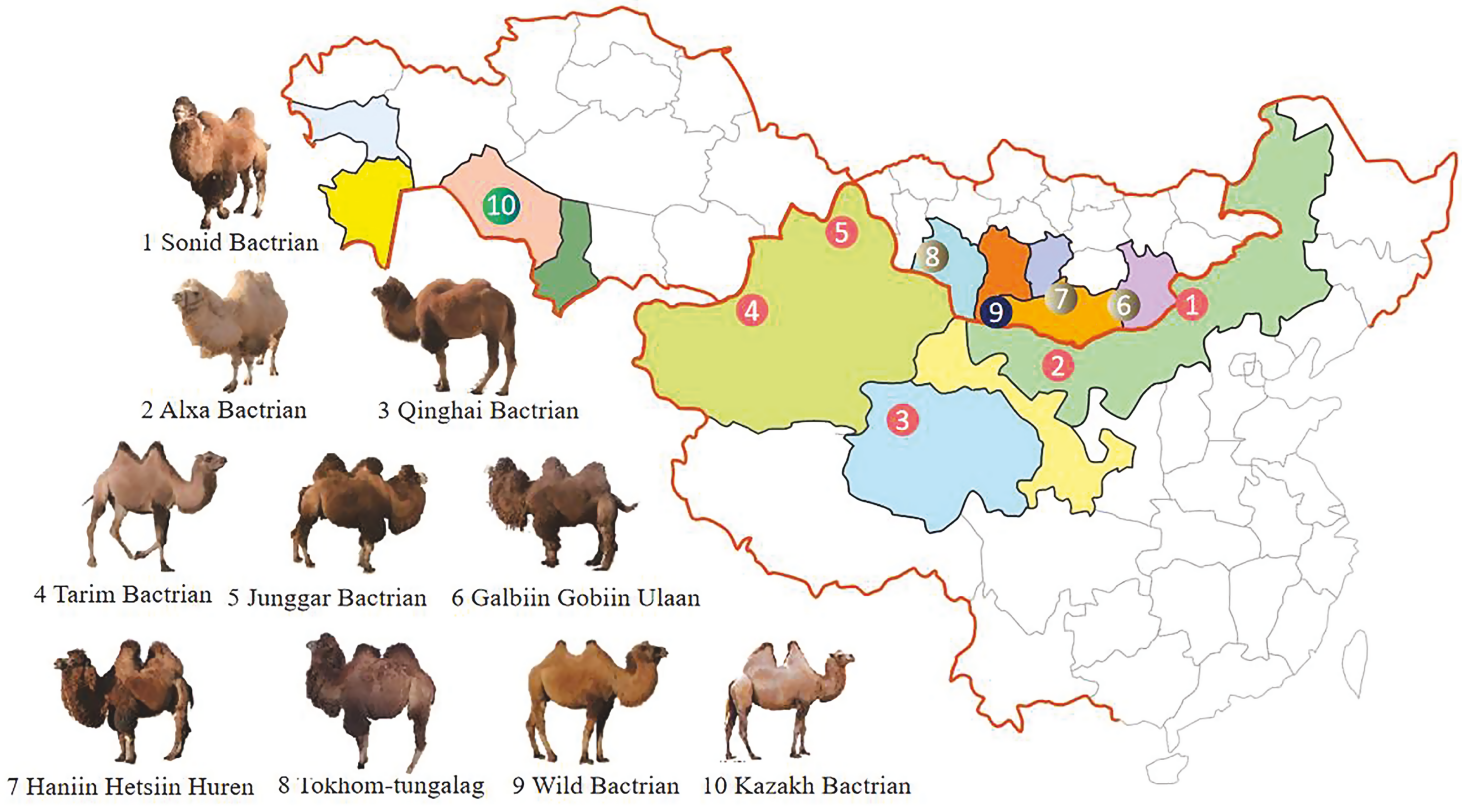
Geographic locations of Bactrian camel breeds from China, Mongolia, and Kazakhstan.

Based on distribution areas and phenotypic characteristics, several different breeds of Bactrian camel exist. In China, Bactrian camels are mainly distributed across Inner Mongolia, Xinjiang, Qinghai, and Gansu and are classified into five breeds: Alxa, Sonid, Qinghai, Tarim, and Junggar Bactrian camel (Jirimutu et al., 2009). Mongolian domestic Bactrian camels are mainly distributed in Umnegobi, Dornogobi, Dundgobi, Bayankhongor, and Gobi-Altai, and three breeds are recognized: Galbiin Gobiin Ulaan (GGU), Haniin Hetsiin Huren (HHH), and Tokhom-tungalag or Hos Zogdort (HZ) (Chuluunbat et al., 2014). In terms of camel breeding in Kazakhstan, camel breeds, such as Kazakh, Mongolian, and Kalmyk Bactrian camels are mainly bred and found in Almaty, Batysdykazakstan, Ongtustikkazakstan, and Aktube (Elmira et al., 2020). The most common locations and characteristics of Bactrian camel breeds are listed in Figure 3 and Table 1.

**Table 1.** Bactrian camel breeds in China, Mongolia, and Kazakhstan

Country	Breed	Distribution	Coat color	Potential use
China	Alxa	Alxa, Badain Jaran, and Tengger desert, Inner Mongolia	Brown, red, yellow, white	Multipurpose camel
	Sonid	Xilingol league of Inner Mongolia	Brownish, red-based, apricot yellow, brown	Multipurpose camel
	Qinghai	Ulan county, Dulan county, and Haixi Mongolian and Tibetan Autonomous Prefecture of Qinghai province	Light brown, red, gray, and white	Multipurpose camel
	Tarim	Xinjiang Uygur Autonomous Region and Tianshan mountain desert	Brown, reddish-brown, yellow, red	Multipurpose camel
	Junggar	Altai city, Tarbagatay city, and Mulei county, Xinjiang Uygur Autonomous Region	Brown, yellow	Multipurpose camel
Mongolia	Galbiin Gobiin Ulaan	Khanbogd and Bayan-ovoo soum of Umnugobi province	Brownish red	Multipurpose camel
	Haniin Hetsiin Huren	Mandal-ovoo soum of Umnugobi province	Brownish red	Multipurpose camel
	Tokhom-tungalag/Hos Zogdort	Togrog Soum of Gobi-Altai province	Brown	Multipurpose camel
Kazakhstan	Kazakh Bactrian	South and West Kazakhstan province, Almaty province	Brown, gray, and white	Dairy camel
China/Mongolia	Wild two-humped camel	Taklamakan Desert, Gashun Gobi Desert, and Arjin Mountains in the Lop Nur Lake region, Great Gobi Strictly Protected Area “A”	Light brown	None

In addition, wild two-humped camels were discovered by Nikolaj Przewalski in 1878 (Peters and Driesch, 1997). As early as the 18th century, wild two-humped camels were widely distributed in many areas, such as Central Asia, reaching the Yellow River Bay in the East, the Qinghai Tibet Plateau in the south, the Caspian Sea in the West, and Baikal Lake in the north. The distribution area was continuous, and the total population was more than 10,000. In the early 1980s, investigations and estimations determined that there were 2,500 to 3,000 wild two-humped camels (Tulgat and Schaller, 1992). However, due to the impact of human activities and the deteriorating ecological environment, the distribution area of wild two-humped camels reduced, and the population declined. Nowadays, their range is limited to only four locations worldwide: three areas in China (Taklamakan Desert, Gashun Gobi Desert, and Arjin Mountains in the Lop Nur Lake region) and one in Mongolia (Great Gobi Strictly Protected Area “A”). At present, their total population was only 900 to 1,600, less than that of the giant panda (Yadamsuren et al., 2019).

## Evolution History and Domestication of Bactrian Camel

### Evolution history of Bactrian camel

Although Bactrian camels are mainly distributed in Asia, their ancestors can be traced back to the *Protylopus* in the North American savannah during the Eocene period (~45 million years ago), measuring about 80 cm long and weighing 2.6 kg, with no hump, resembling the current hare (Rybczynski, 2013). In the Oligocene period (33.9 to 23 million years ago), camelids evolved rapidly. Named *Poebrotherium*, they lost two side toes, resembling the two-toed structure of modern camels. They still had no hump and stood about three feet tall, about the size of today’s deer. After the Oligocene period, the two-toed *Protylopus* gradually evolved into several genera of camelids, one of which evolved into the *Procamelus* during the Oligocene to Miocene period (20 to 5 million years ago). During this period, the Earth environment began to become dry and cold, and grasslands were replaced by sand. With the change in the environment, the *Procamelus* evolved a unique tooth and jaw structure similar to modern camels and had a similar pace as modern camels. During the Pliocene period (2.5 million years ago), North America and South America were connected, which brought opportunities for the migration of the *Paracamelus*, and the progenitors of Lamini entered South America around 3 million years ago (Rybczynski, 2013; Monchot, 2015). The others crossed the Bering Isthmus to Asia and evolved into dromedaries and Bactrian camels during the Late Miocene period (7.24 to 4.9 million years ago) (Honey et al., 2005). The divergence time between the Bactrian camel and dromedary was ~4.4 million years ago (1.9 to 7.2 million years ago) (Wu et al., 2014), and genetic studies confirmed (Ji et al., 2009; Silbermayr et al., 2010; Jirimutu et al., 2012; Mohandesan et al., 2017) that the wild two-humped camel was different from the domesticated camel, and the divergent time was estimated as 0.43 million years ago (95% confidence interval: 0.13 to 0.73 million years ago) by whole-genome sequencing (Ming et al., 2020a), which was slightly later than that based on mtDNAs (0.7 or 1.1 million years ago) (Ji et al., 2009; Mohandesan et al., 2017).

### Domestication of Bactrian camels

Based on the geological fossil data found by paleontologists (Reitz and Wing, 2008), the period of Bactrian camel domestication was estimated as 4,500 years ago, and the evidence related to domestication regions was mainly concentrated in Northern Iran and Southern Turkmenistan (Bulliet, 1975). However, some scholars believed that in addition to Iran and Turkmenistan, the domestication areas of Bactrian camels also included southern Kazakhstan, Western Mongolia, and Northern China, mainly based on the existence of wild two-humped camels in these areas (Peters and Driesch, 1997). For a long time, according to the individual morphological and anatomical characteristics of domestic Bactrian camel and the extant wild two-humped camel, it was speculated that the domestic Bactrian camel was domesticated from the extant wild two-humped camel. Nevertheless, molecular genetics research based on mtDNAs (Ji et al., 2009; Mohandesan et al., 2017) and Y chromosomes (Felkel et al., 2019) discovered dramatic sequence variations between domestic and the wild two-humped camel, suggesting that they were not the same subspecies, at least in their maternal origins.

Furthermore, the haplotypes of skeletal mitochondrial partial sequences of 12 Bactrian camel bones from Late Bronze and Early Iron Age sites found in Uzbekistan and Syria were shared with modern domestic Bactrian camels (Trinks et al., 2012) but different from those of extant wild two-humped camels, suggesting only one domestication event; that is, the wild two-humped camel was a separate lineage. Another possible place of origin was Iran, where early skeletal remains of domestic Bactrian camels (around 2500 to 3000 BC) were discovered (Yam and Khomeiri, 2015). However, incomplete archaeological findings provided little conclusive information about the actual domestication history of Bactrian camels.

**Figure 4.**
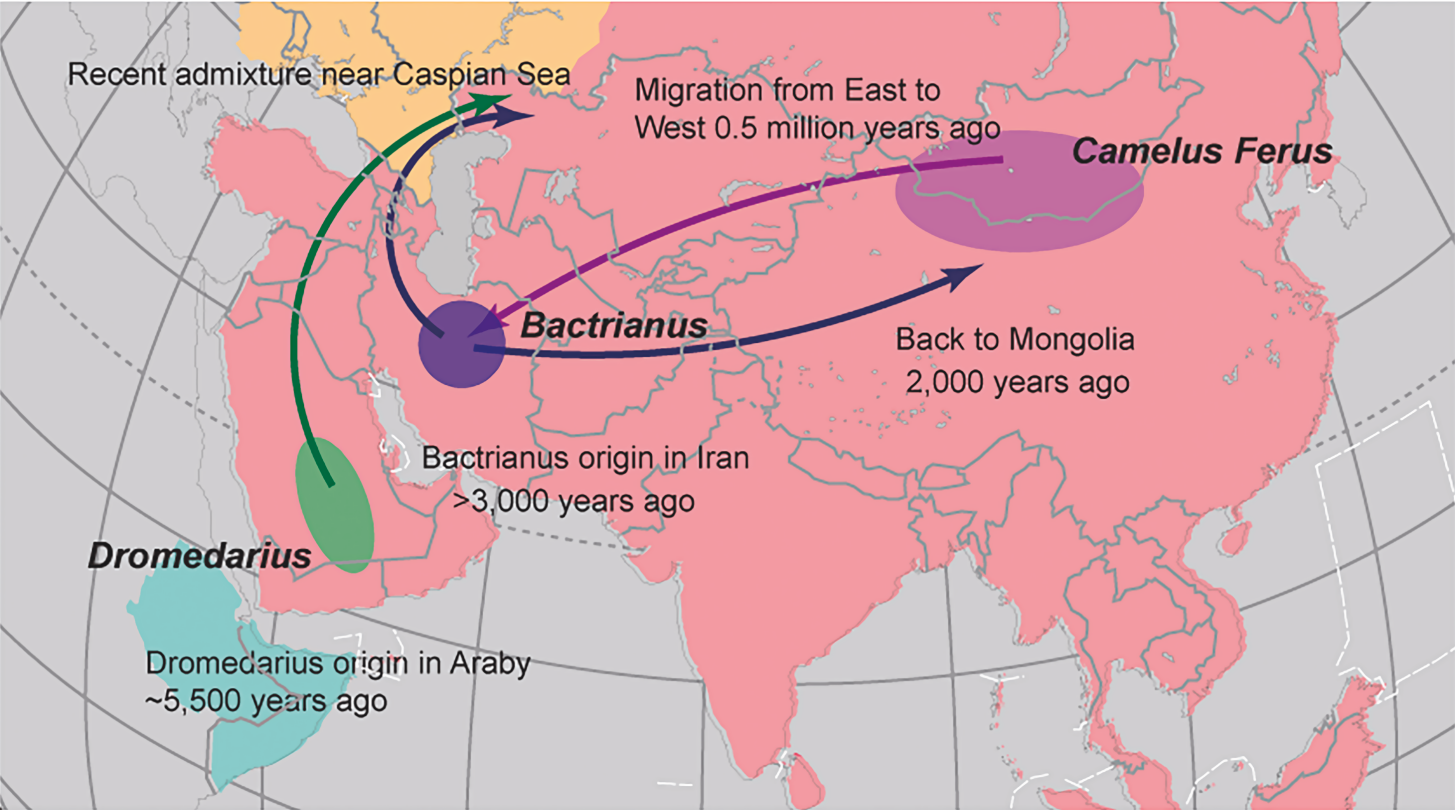
A proposed migration route of Bactrian camels. Reprinted from the Supplementary Material of Ming et al. (2020a).

Recently, the whole-genome sequencing including domestic and the wild two-humped camels were used to reveal the origin, migration, and domestication of Bactrian camel (Ming et al., 2020a). A significantly higher genetic diversity and divergence were displayed in the Iranian camels, and the earliest branching among the domestic Bactrian camels occurred between Iran and all the other Bactrian camel populations. Although evident introgression of dromedaries was observed in Central Asia (11,711 non-overlapping 100 kb segments, *Z*-score >2) by using the “BABA/ABBA,” the study demonstrated that it will not influence the results by removing the introgressed genomic segments (Ming et al., 2020a). Therefore, it is valid to assume that Bactrian camels were first domesticated in Central Asia less than 4.45 thousand years ago, and then migrated back to East Asia around 2.40 thousand years ago (Figure 4).

### Characteristics of Bactrian camel genome

While the Bactrian camel made a great contribution to transportation on the Silk Road and could be portrayed as a bridge between Eastern and Western cultures, little is known about the camel genome. The first draft of the Bactrian camel genome was completed in 2012 (Jirimutu et al., 2012). The estimated size of the Bactrian camel genome was 2.38 Gb, close to the camel genome size (2.02 to 2.40 Gb) calculated based on haploid DNA contents (C values). They predicted 20,821 protein-coding genes, averaging eight exons and a 1322-bp coding region per gene. Based on phylogenetic analysis, the authors also revealed that the camel shared a common ancestor with even-toed ungulates around 55 to 60 million years ago. Rapidly evolving genes were significantly enriched in metabolic pathways, perhaps helping Bactrian camels optimize their energy storage and production in the desert. The olfactory receptors were enriched in lower heterozygosity genomic regions in the domestic camel. After annotation, the results also suggested that the specific cytochrome P450 families and unusual immune system were useful for survival in the desert (Jirimutu et al., 2012). Subsequently, a male Bactrian camel’s genome was de novo assembled (Burger and Palmieri, 2014), and the genome size reached 1.6 Gb. More than 116,000 heterozygous SNPs were detected, and the genome-wide nucleotide diversity was similar to that of other domesticated ungulates (cattle and pig) based on average 6.6-fold coverage (Burger and Palmieri, 2014).

A similar report related to sequencing the genome of Bactrian camel, dromedary, and alpaca was published in 2014 (Wu et al., 2014). The estimation of the Bactrian camel genome size reached 2.45 Gb, predicting the presence of 20,251 genes, similar to the earlier report (Jirimutu et al., 2012). Evolutionary analysis found that the Bactrian camel and the dromedary had higher non-synonymous/synonymous (Ka/Ks) substitution ratios (ω-values), which raised the possibility of Bactrian camels adapting to the harsh desert environment. Comparative genomics analyses also revealed complex features related to desert adaptations, including fat and salt metabolism, stress responses to arid and hot environments, and intense ultraviolet radiation. Transcriptomic analysis of Bactrian camels further showed unique osmoregulation, osmoprotection, and compensatory mechanisms for water reservation underpinned by high blood glucose levels (Wu et al., 2014).

A perfect assembly report, related to high-coverage long-read sequencing and chromatin interaction mapping of wild two-humped camel genome, was completed in 2020 (Ming et al., 2020b). This analysis assembled the most contiguous Bactrian camel genome to date, and exhibited remarkable improvement over existing short-read assemblies of the same species. The contig N50 reached 5.37 Mb and the scaffold N50 was 76.03 Mb. The authors also illustrated that immunoglobulin and T-cell receptor gene loci could be uniquely localized to specific chromosomes, and the classical major histocompatibility complex region was resolved into a single contig without any gaps. Genomic comparison of Bactrian camels from different research was shown in Table 2.

**Table 2.** Genomic comparison of Bactrian camels from different research

Species	Location	Sequencing platform	Sequencing depth (×)	Genome size (Gb)	Contig N50 (kb)	Scaffold N50 (Mb)	Gene number
Wild two-humped camel^*a*^	Mongolian Wild Camel Protection Area, Mongolia	Illumina Solid 3 Roche	76	2.01	90.3	2.0	20821
Domestic Bactrian camel^*b*^	Inner Mongolia, China	Illumina HiSeq 2000	79.3	1.99	139.0	8.8	20251
Domestic Bactrian camel^*c*^	Austrian Zoo Herberstein	Illumina	6.56	1.57	2.8	–	–
Wild two-humped camel^*d*^	Great Gobi Strictly Protected Area “A”, Mongolia	Pacific Biosciences Sequel	130	2.09	5.4	76.0	–

^*a*^See Jirimutu et al. (2012).

^*b*^See Wu et al. (2014).

^*c*^See Burger and Palmieri (2014).

^*d*^See Ming et al. (2020).

## Genetic Diversity of Bactrian Camel Genome

### Genetic diversity based on the mtDNA

The mitochondrial genome (mtDNA) has the characteristics of simple structure, maternal inheritance, a high evolution rate, and no recombination and has been used as a genetic marker of genetic diversity in livestock. The first molecular evolutionary analysis of the family Camelidae described by the mitochondrial cytochrome b (Cytb) gene (Stanley et al., 1994) revealed that the divergence time of Old Word (Camelini) and New World (Lamini) tribes was consistent with the fossil record. This opened the door for researchers to explore the ancestral evolution of Camelidae. Jianlin et al. (1999) analyzed the mitochondrial genome of wild two-humped and domestic Bactrian camels by restriction fragment length polymorphism (RFLP), which showed that there were differences in enzyme digestion bands at three endonuclease positions between them. In addition, high level of mitochondrial sequence divergence (2.4%) between domestic Bactrian and wild two-humped camels had been revealed using the sequence of mitochondrial Cytb gene (Ji et al., 2009). Meanwhile, using an 804-bp mitochondrial fragment, the authors reported that there were eight haplotypes (six domestic camel haplotypes and two wild two-humped camel haplotypes) in wild two-humped, hybrid, and domestic Bactrian camels originating from Mongolia, China, and Austria and found high sequence divergence (1.9%) between wild two-humped and domestic Bactrian camels (Silbermayr et al., 2010).

In different domestic breeds, researchers analyzed the genetic diversity and population structure based on the different mitochondrial segments. The genetic diversity and phylogeographic structure were detected based on the 809-bp mtDNA fragment (Ming et al., 2017) and the Cytb gene (Ming et al., 2016) in 11 domestic Bactrian populations from different locations, including China, Mongolia, and Russia, and one wild two-humped camel group from Mongolia. The domestic and wild two-humped camels had two distinct lineages, and the wild two-humped camels displayed lower levels of nucleotide and haplotype diversity. The levels of nucleotide and haplotype diversity within Mongolia were slightly higher than those of camels from China and Russia; however, there was no significant genetic divergence among different geographical locations, which suggests a strong gene flow due to the wide movement of domestic Bactrian camels (Ming et al., 2016, 2017). Subsequently, research based on the mitochondrial ATP8 and ATP6 genes also confirmed this standpoint (Yi et al., 2017). However, research by Chen et al. (2019) showed that there existed at least two maternal lineages of domestic Bactrian camels, and the divergence time of them was estimated as 165 thousand years ago (95% credibility interval 117 to 222 thousand years ago), indicating that several different evolutionary lineages were incorporated into the domestic gene pool during the initial domestication process.

**Figure 5.**
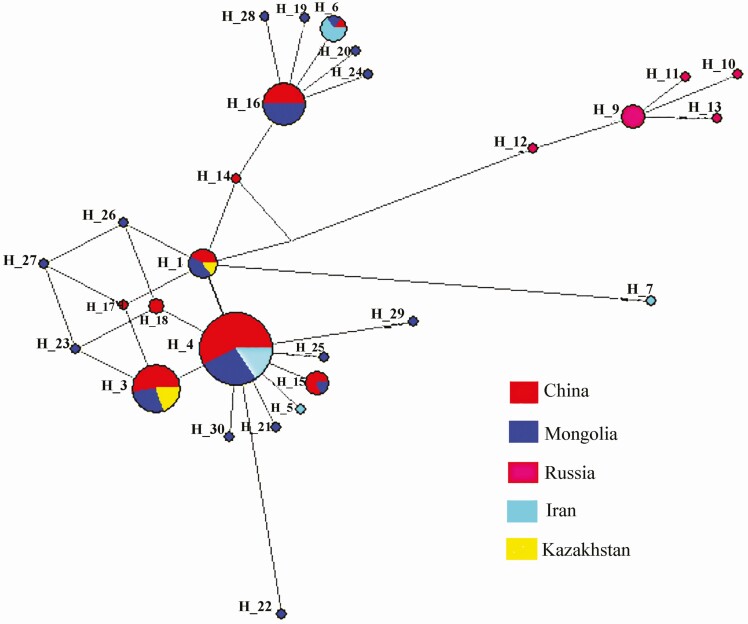
Median-joining network for domestic Bactrian camel population haplotypes from China, Mongolia, Russia, Iran, and Kazakhstan. The areas of the circles were proportional to the haplotype frequencies. Haplotypes of each population from different geographic regions, such as China, Mongolia, Iran, and Kazakhstan, did not cluster together according to their geographic regions. Reprinted from the Material of Ming et al. (2021).

Recently, a comprehensive study was completed to reveal genetic diversity by mtDNA control region sequences of 182 individuals from Old World camels (Ming et al., 2021). Thirty-two haplotypes were confirmed in Bactrian camels, including domestic and wild two-humped camels, while 14 haplotypes were defined in dromedaries. The wild two-humped camel showed the lowest haplotype (π = 0.00115) and nucleotide diversity, while the dromedaries investigated had the highest (π = 0.02323). Meanwhile, the phylogenetic relationship among domestic Bactrian camel population haplotypes was shown that haplotypes of each population from different geographic regions (China, Mongolia, Iran, and Kazakhstan) did not cluster together according to their geographic regions (Figure 5), and there were several shared haplotypes (H_1, H_3, H_4, and H_20) between them. It can be predicted domestic Bactrian camels frequently traveled between different geographic regions. Moreover, haplotypes specific to a geographic region were also found, such as five haplotypes (H_9 to H_14) only represented in Russia Bactrian camels; H_5 and H_7 only found in Iran Bactrian camels; haplotypes H14, H17, and H18 were only discovered in Chinese Bactrian camels, and there were 12 haplotypes were only found in Mongolian Bactrian camels (Ming et al., 2021).

In Mongolian domestic breeds, researchers traced possible differentiation within and between the three local breeds (HHH, HZ, GGU) and the common Mongolian native camel (MNT) (Chuluunbat et al., 2014). The nucleotide and haplotype diversity were lower in GGU than in HHH and HZ breeds, and some haplotypes were shared by individuals in the three breeds and one native population. The mean haplotype diversity (Hd = 0.755) was lower than that of Ming et al.’s study (Hd = 0.796) (Ming et al., 2017).

### Genetic diversity based on the nuclear genome

A previous study focused on the genetic diversity of Bactrian camels from China and Mongolia. The population genetic diversity was studied on 254 Bactrian and 5 wild two-humped camels from China and Mongolia using genotype data from 16 microsatellite markers (Gao et al., 2008). The analysis of genetic distances and correlation cluster showed that there was no significant genetic differentiation in the domestic Bactrian camels between China and Mongolia, however, there was significant genetic differentiation between Bactrianus and wild two-humped camels (Gao et al., 2008). In addition, Wang (2009) reported that there existed rich genetic diversity among five domestic Bactrian camel populations from China based on 10 microsatellite markers (Wang, 2009). In order to investigate the genetic diversity, relationship, differentiation, and possible inbreeding at the molecular level, 452 Bactrian camel individuals (9 Chinese camel populations and 1 Mongolian camel population) from China and Mongolia were genotyped using 18 microsatellite markers (Xiaohong, 2011). All the Bactrian camel populations had rich genetic diversity, and the genetic differentiation among the 10 populations was highly significant (*P* < 0.001) with 9.6% of the total genetic variance present among the populations and the remaining 90.4% among individuals within the populations (Xiaohong, 2011). Chuluunbat et al. (2014) investigated the genetic diversity in Mongolian Bactrian camels using nuclear markers and found levels of genetic diversity in Mongolian populations similar to those reported for Chinese Bactrian camels. Little differentiation was detected between single breeds, except for a small group originating from the northwestern Mongolian Altai region (Chuluunbat et al., 2014).

### Genetic diversity based on the whole-genome sequencing

**Figure 6.**
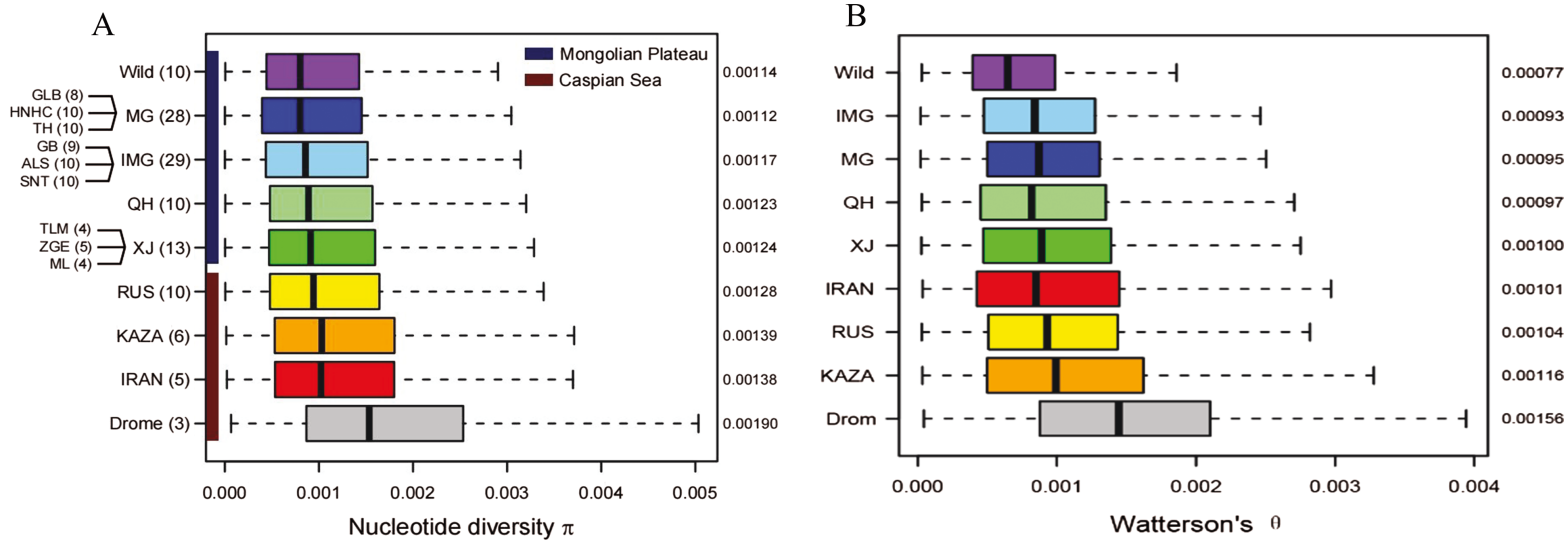
The pairwise nucleotide diversity π (A) and Watterson’s θ (B) of the camel populations. Reprinted from the Material of Ming et al. (2020a).

Similar to its late domestication history, the Bactrian camel is one of the last livestock animals to have its population genome sequenced and genetic diversity revealed. The domestic Bactrian camels were chosen to cover as many major geographic regions as possible, including China, Mongolia, Kazakhstan, Russia, and Iran and compared to the dromedary from Iran (Ming et al., 2020a). The pairwise nucleotide diversity π of dromedaries (1.54 × 10^−3^) was significantly higher than that of Bactrian camels from all geographic regions (0.88 × 10^−3^ to 1.11 × 10^−3^); among the Bactrian camels, the wild two-humped camels showed the lowest π (0.88 × 10^−3^) compared with all the domestic populations, consistent with the previous nucleotide diversity estimates based on mitochondrial genomes (Ming et al., 2016, 2017). In addition, the domestic populations living in Central Asia generally showed higher diversity (1.03 × 10^−3^ to 1.11 × 10^−3^) than those living in East Asia (0.95 × 10^−3^ to 1.02 × 10^−3^). The tendency was also confirmed by Watterson’s θ (Figure 6).

## Conclusion

The Bactrian camel is a key species in the Gobi Desert, and it is also a unique large livestock animal living in desert and semi-desert environments around the world. Because most of the distribution areas of Bactrian camels are areas where ethnic minorities gather, Bactrian camels have been closely linked to ethnic minorities for thousands of years, playing an irreplaceable role in the reproduction and survival of ethnic minorities. The Bactrian camel has a special status among mammals domesticated by humans since they are highly adapted to the extreme desert ecosystem. It is a multipurpose animal used for dairy production, racing, and transportation. China, Mongolia, Russia, and Kazakhstan are the countries with the largest populations of Bactrian camels in the world. It is imperative to comprehensively and systematically study the genetic diversity, phylogeny, population differentiation, and inbreeding of Bactrian camels in different regions at the molecular level, so as to provide a scientific basis for the protection of Bactrian camel germplasm resources and variety cultivation.

## About the Authors

**Liang Ming** graduated from Inner Mongolia Agricultural University and obtained her PhD degree in 2016 in agricultural product processing and storage engineering. She is working in the College of Food Science and Engineering at Inner Mongolia Agricultural University as a lecturer. Her research interests are focused on Bactrian camel genome, including origin, evolution, genetic diversity, and healthcare benefits of Bactrian camel milk for liver disease. The latest publication (2020) in Molecular Ecology Resources constructed a chromosome-level assembly of wild camel genome, and the publication in Communications Biology investigates the Central Asian origin of domestic Bactrian camels and a roughly west-to-east route of migration back to the Mongolian Plateau.

**Dalai Siren** is a PhD student in the College of Food Science and Engineering at Inner Mongolia Agricultural University, China, majoring in food science. He obtained his master’s degree in 2016 in Food Science and Engineering. He is the office director of Inner Mongolia Institute of Camel Research. His research interests are focused on Bactrian camel genome, Bactrian camel meat, Bactrian camel milk, and so on.

**Surong Hasi** is a professor in the College of Veterinary Medicine of Inner Mongolia Agricultural University, graduated from China Agricultural University, and obtained his PhD degree in 2004 in domestic animal parasitology. He completed his post-doc research in 2008 at Leuven University in Belgium and worked as visiting scholar at the LCH laboratory in France and the Texas A&M University in the United States, respectively. He is the vice president of the Chinese Camel Industry Association, the chairman of Camel Protection Association of Inner Mongolia, and the executive director of the Chinese Society of Veterinary Pharmacology and Toxicology. His research interests are focused on parasitic diseases of domestic ruminants, biological characteristics of Bactrian camel and camel diseases, drug–drug interactions, and pharmacokinetics of drugs in domestic animals.

**Tuyatsetseg Jambl** is a professor and dean of Mongolian University of Science and Technology, School of industrial technology. She graduated from Moscow State University of Food Production, Russia in 1988. She worked at Talkh Chikher Company until 1990 as an engineer. She obtained her PhD degree from Mongolian National University of Science and Technology in 2008. From 2009 to 2014, she was dean of the School of Food Engineering and Biotechnology, National University of Science and Technology of Mongolia.

**Rimutu Ji** is a professor, working in the College of Food Science and Engineering at Inner Mongolia Agricultural University, China. He graduated from this University in 1988 and then obtained his PhD degree in 2007 in Bactrian camel milk nutrition. In 2000, he started investigating the genetic diversity, origin, and process of domestication in Bactrian camels. In 2012, he completed the mapping and deciphering of the world’s first wild camel genome sequences. In 2014, he founded the Inner Mongolia Camel Research Institute and was dedicated to studying the Chinese Bactrian camel genome and milk nutrition. In 2016, he founded an internationally advanced biopolymer application joint laboratory in Ulaanbaatar, Mongolia, focusing on Mongolian Bactrian camel genome. In 2017, he was selected as a member of the professional committee of horses, donkeys, and camels. He won the “Russian Ministry of Agriculture Natural Science Award” and “Shen Nong Chinese Agricultural Science and Technology Award”.

## References

[CIT0001] Al-Ali, A., H. Husayni, and D.A. Power. 1988. Comprehensive biochemical analysis of the blood of the camel (*Camelus dromedarius*). Comp. Biochem. Physiol. B. 89:35–37. doi:10.1016/0305-0491(88)90257-X 3356128

[CIT0002] Bouâouda, H., M.R. Achâaban, M. Ouassat, M. Oukassou, M. Piro, E. Challet, K.E. Allali, and P. Pévet. 2014. Daily regulation of body temperature rhythm in the camel (*Camelus dromedarius*) exposed to experimental desert conditions. Physiol. Rep. 2:1–16. doi:10.14814/phy2.12151 PMC4270234 25263204

[CIT0003] Bulliet, R . 1975. The camel and the wheel. New York (NY): Columbia University Press; p. 327.

[CIT0004] Burger, P.A., and N. Palmieri. 2014. Estimating the population mutation rate from a *de novo* assembled Bactrian camel genome and cross-species comparison with dromedary ESTs. J. Hered. 105:839–846. doi:10.1093/jhered/est005 23454912 PMC4201309

[CIT0005] Chen, S.G., J. Li, F. Zhang, B. Xiao, J.M. Hu, Y.Q. Cui, M. Hofreiter, X.D. Hou, G.L. Sheng, X.L. Lai, et al. 2019. Different maternal lineages revealed by ancient mitochondrial genome of *Camelus bactrianus* from China. Mitochondrial DNA A DNA Mapp. Seq. Anal. 30(7):786–793. doi:10.1080/24701394.2019.1659250 31542986

[CIT0006] Chuluunbat, B., P. Charruau, K. Silbermayr, T. Khorloojav, and P.A. Burger. 2014. Genetic diversity and population structure of Mongolian domestic Bactrian camels (*Camelus bactrianus*). Anim. Genet. 45:550–558. doi:10.1111/age.12158 24749721 PMC4171754

[CIT0007] Elmira, A., A. Nuradin, A. Svitojus, and A. Galymzhan. 2020. Genetic typing of South Kazakhstan populations’ dairy camels using DNA technology. Anim. Biotechnol. 31:547–554. doi:10.1080/10495398.2019.1669625 31564209

[CIT0008] Emmanuel, B., and A. Nahapetian. 1980. Fatty acid composition of depot fats, and rumen wall of the camel (*Camelus dromedarius*). Comp. Biochem. Physiol. B. 67:701–704. doi:10.1016/0305-0491(80)90435-6

[CIT0009] Faye, B., and G. Konuspayeva. 2012. The encounter between Bactrian and dromedary camels in Central Asia. In: Knoll, E.-M. and P.A. Burger, editors. Camels in Asia and North Africa: interdisciplinary perspectives on their past and present significance. Vienna (Austria): Austrian Academy of Sciences Press; p. 27–33.

[CIT0010] Felkel, S., B. Wallner, B. Chuluunbat, A. Yadamsuren, B. Faye, G. Brem, C. Walzer, and P.A. Burger. 2019. A first Y-chromosomal haplotype network to investigate male driven population dynamics in domestic and wild Bactrian camels. Front. Genet. 10:423. doi:10.3389/fgene.2019.00423 31178891 PMC6537670

[CIT0011] Gao, H.W., J. Wang, J.X. He, L.Y. Chen, R. Ji, and H. Meng. 2008. Analysis on the genetic diversity and evolution in the *Camelus bactrianus* using SSR markers. J. Shanghai Jiaotong Univ. (Sci.). 27:89–95.

[CIT0012] Hamers-Casterman, C., T. Atarhouch, S. Muyldermans, G. Robinson, C. Hammers, E. Songa, N. Bendahman, and R. Hammers. 1993. Naturally occurring antibodies devoid of light chains. Nature. 363:446–448. doi:10.1038/363446a0 8502296

[CIT0013] Hare, J . 2008. *Camelus ferus*. The IUCN red list of threatened species [Internet]. Version 2018.2; e.T63543A12689285 [species assessed June 30, 2008; page accessed February 19, 2019]. doi:10.2305/IUCN.UK.2008.RLTS.T63543A12689285.en

[CIT0014] Hasi, S., J. Yao, S. Yu, and Y. Tian. 2018. Diversity and distribution of CYP gene family in Bactrian camel. Funct. Integr. Genomics. 18:23–29. doi:10.1007/s10142-017-0571-y 28900766 PMC5748438

[CIT0015] Honey, J.G., J.A. Harrison, D.R. Prothero, and M.S. Stevens. 2005. Evolution of tertiary mammals of North America: volume 1, terrestrial carnivores, ungulates, and ungulate like mammals. New York, Cambridge: Cambridge University Press; p. 439–461.

[CIT0016] Ji, R., P. Cui, F. Ding, J. Geng, H. Gao, H. Zhang, J. Yu, S. Hu, and H. Meng. 2009. Monophyletic origin of domestic bactrian camel (*Camelus bactrianus*) and its evolutionary relationship with the extant wild camel (*Camelus bactrianus ferus*). Anim. Genet. 40:377–382. doi:10.1111/j.1365-2052.2008.01848.x 19292708 PMC2721964

[CIT0017] Jianlin, H., Q. Jiexia, M. Zhenming, Z. Yaping, and W. Wen. 1999. Rapid communication: three unique restriction fragment length polymorphisms of EcoRI, PvuII, and ScaI digested mitochondrial DNA of Bactrian camels (*Camelus bactrianus ferus*) in China. J. Anim. Sci. 77:2315–2316. doi:10.2527/1999.7782315x 10462013

[CIT0018] Jirimutu, R., C. Gang Liang, and Y. Zhenyu. 2009. The Bactrian camel and Bactrian camel milk. Beijing, China: Chinese Light Industry Press; p. 9–12.

[CIT0019] Jirimutu, R., L. Ming, and J. He. 2022. Camel genome and germplasm resources. Beijing, China: China Agricultural Press; p. 123–125.

[CIT0020] Jirimutu, R., Z. Wang, G. Ding, G. Chen, Y. Sun, Z. Sun, H. Zhang, L. Wang, S. Hasi, Y. Zhang, et al. 2012. Genome sequences of wild and domestic Bactrian camels. Nat. Commun. 3:1202. doi:10.1038/ncomms2192 23149746 PMC3514880

[CIT0021] Ming, L., D. Siren, L. Yi, L. Hai, J. He, and J. Rimutu. 2021. Mitochondrial DNA variation and phylogeography of Old World camels. Anim. Biosci. 34:525–532. doi:10.5713/ajas.20.0319 32898955 PMC7961272

[CIT0022] Ming, L., Z. Wang, L. Yi, M. Batmunkh, T. Liu, D. Siren, J. He, N. Juramt, T. Jambl, Y. Li, et al. 2020b. Chromosome-level assembly of wild Bactrian camel genome reveals organization of immune gene loci. Mol. Ecol. Resour. 20:770–780. doi:10.1111/1755-0998.13141 32012460

[CIT0023] Ming, L., L. Yi, F.C. Guo, S. Siriguleng, and J. Jirimutu. 2016. Molecular phylogeny of the Bactrian camel based on mitochondrial Cytochrome b gene sequences. Genet. Mol. Res. 15:1–8. doi:10.4238/gmr.15038983 27706756

[CIT0024] Ming, L., L. Yi, R. Sa, Z.X. Wang, Z. Wang, and R. Ji. 2017. Genetic diversity and phylogeographic structure of Bactrian camels shown by mitochondrial sequence variations. Anim. Genet. 48:217–220. doi:10.1111/age.12511 27775167 PMC5347888

[CIT0025] Ming, L., L. Yuan, L. Yi, G. Ding, S. Hasi, G. Chen, T. Jambl, N. Hedayat-Evright, M. Batmunkh, G.K. Badmaevna, et al. 2020a. Whole-genome sequencing of 128 camels across Asia reveals origin and migration of domestic Bactrian camels. Commun. Biol. 3:1. doi:10.1038/s42003-019-0734-6 31925316 PMC6946651

[CIT0026] Mohandesan, E., R.R. Fitak, J. Corander, A. Yadamsuren, B. Chuluunbat, O. Abdelhadi, A. Raziq, P. Nagy, G. Stalder, C. Walzer, et al. 2017. Mitogenome sequencing in the genus *Camelus* reveals evidence for purifying selection and long-term divergence between wild and domestic Bactrian camels. Sci. Rep. 7:9970. doi:10.1038/s41598-017-08995-8 28855525 PMC5577142

[CIT0027] Monchot, H . 2015. From palaeontology to zooarchaeology, importance of the camels through time and history. In: Konuspayeva, G. editors. Proceedings of 4th Conference of ISOCARD, Almaty, Kazakhstan, June 8-12, 2015. Қазақ университеті Press; p. 43–47.

[CIT0028] Peters, J., and V.D. Driesch. 1997. The two-humped camel (*Camelus bactrianus*): new light on its distribution, management and medical treatment in the past. J. Zool. 242:651–679. doi:10.1111/j.1469-7998.1997.tb05819.x

[CIT0029] Reitz, E.J., and E.S. Wing. 2008. Zooarchaeology. 2nd ed. New York, Cambridge: Cambridge University Press.

[CIT0030] Rybczynski, N., J.C. Gosse, C.R. Harington, R.A. Wogelius, A.J. Hidy, and M. Buckley. 2013. Mid-Pliocene warm-period deposits in the High Arctic yield insight into camel evolution. Nat. Commun. 4:1550. doi:10.1038/ncomms2516 23462993 PMC3615376

[CIT0031] Saipolda, T . 2005. Mongolian camels. In: Cardellino, R., A. Rosati, and C. Mosconi, editors. Current status of genetic resources, recording and production systems in African, Asian and American Camelids. Proceedings of the ICAR/FAO Seminar, Sousse, Tunisia, May 30, 2004. Rome (Italy): ICAR; p. 73–79.

[CIT0032] Silbermayr, K., P. Orozco-terWengel, P. Charruau, D. Enkhbileg, C. Walzer, C. Vogl, F. Schwarzenberger, P. Kaczensky, and P.A. Burger. 2010. High mitochondrial differentiation levels between wild and domestic Bactrian camels: a basis for rapid detection of maternal hybridization. Anim. Genet. 41:315–318. doi:10.1111/j.1365-2052.2009.01993.x 19968638

[CIT0033] Stanley, H.F., M. Kadwell, and J.C. Wheeler. 1994. Molecular evolution of the family Camelidae: a mitochondrial DNA study. Proc. Biol. Sci. 256:1–6. doi:10.1098/rspb.1994.0041 8008753

[CIT0034] Trinks, A., P. Burger, N. Benecke, and J. Burger. 2012. Ancient DNA reveals domestication process: the case of the two-humped camel. In: Knoll, E.-M., and P.A. Burger, editors. Camels in Asia and North Africa: interdisciplinary perspectives on their past and present significance. Vienna (Austria): Austrian Academy of Sciences Press; p. 79–86.

[CIT0035] Tulgat, R., and G.B. Schaller. 1992. Status and distribution of wild Bactrian camels (*Camelus bactrianus ferus*). Biol. Conserv. 62:11–19. doi:10.1016/0006-3207(92)91147-K

[CIT0036] Wang, L . 2009. Study on the genetic diversity of domesticated Bactrian camels in Chinese five areas using Microsatellite analyses [master’s thesis]. Xianyang (China): Northwest A&F University.

[CIT0037] Warda, M., A. Prince, H.K. Kim, N. Khafaga, T. Scholkamy, R.J. Linhardt, and H. Jin. 2014. Proteomics of old world camelid (*Camelus dromedarius*): better understanding the interplay between homeostasis and desert environment. J. Adv. Res. 5:219–242. doi:10.1016/j.jare.2013.03.004 25685490 PMC4294715

[CIT0038] Wu, H., X. Guang, M.B. Al-Fageeh, J. Cao, S. Pan, H. Zhou, L. Zhang, M.H. Abutarboush, Y. Xing, Z. Xie, et al. 2014. Camelid genomes reveal evolution and adaptation to desert environments. Nat. Commun. 5:5188. doi:10.1038/ncomms6188 25333821

[CIT0039] Xiaohong, H . 2011. Diversity, phylogenetic relationship and mtDNA heteroplasmy of major Bactrian camel populations on China [PhD thesis]. Beijing (China): Chinese Academy of Agricultural Sciences.

[CIT0040] Yadamsuren, A., O. Daria, and S. Liu. 2019. The seasonal distribution of wild camels (*Camelus ferus*) in relation to changes of the environmental conditions in Mongolia. J. Ecol. 9:293–314. doi:10.4236/oje.2019.98021

[CIT0041] Yam, B.A.Z., and M. Khomeiri. 2015. Introduction to camel origin, history, raising, characteristics, and wool, hair and skin: a review. Int. J. Res. Innov. Earth Sci. 4:496–508. http://www.apexjournal.org

[CIT0042] Yi, L., Y. Ai, L. Liang, L. Hai, J. He, F. Guo, X. Qia, and R. Ji. 2017. Molecular diversity and phylogenetic analysis of domestic and wild Bactrian camel populations based on the mitochondrial ATP8 and ATP6 genes. Livest. Sci. 199:95–100. doi:10.1016/j.livsci.2017.03.015

